# Vitamin D and COVID-19: An Overview of Recent Evidence

**DOI:** 10.3390/ijms221910559

**Published:** 2021-09-29

**Authors:** Drishti Ghelani, Simon Alesi, Aya Mousa

**Affiliations:** Monash Centre for Health Research and Implementation (MCHRI), Public Health and Preventive Medicine, Faculty of Medicine, Nursing and Health Sciences, Monash University, Melbourne, VIC 3168, Australia; drishti.ghelani@monash.edu (D.G.); simon.alesi@monash.edu (S.A.)

**Keywords:** vitamin D, coronavirus, COVID-19, *SARS-CoV-2*, immunity, infection, review

## Abstract

The novel coronavirus severe acute respiratory syndrome (*SARS-CoV-2*) has progressed rapidly from an outbreak to a global pandemic, with new variants rapidly emerging. Coronavirus disease 2019 (COVID-19), the disease resulting from *SARS-CoV-2* infection, can lead to multiorgan damage. Due to the extremely contagious and fatal nature of the virus, it has been a priority of medical research to find effective means of treatment. Amid this search, the role of vitamin D in modulating various aspects of the innate and adaptive immune system has been discussed. This review aims to consolidate the research surrounding the role of vitamin D in the treatment and prevention of COVID-19. While there are some conflicting results reported, the consensus is that vitamin D has a host of immunomodulatory effects which may be beneficial in the context of COVID-19 and that low levels of vitamin D can result in dysfunction of crucial antimicrobial effects, potentially contributing to poor prognosis. Studies also show that the effects of low vitamin D can be mitigated via supplementation, although the benefits of vitamin D supplementation in the treatment of COVID-19 remain controversial.

## 1. Introduction

The coronavirus severe acute respiratory syndrome outbreak of 2019, subsequently named *SARS-CoV-2*, was first reported in Wuhan, China [1]. This coronavirus, initially mimicking symptoms from previous outbreaks such as the Middle Eastern respiratory syndrome (MERS-CoV) and SARS-CoV, is a droplet-borne respiratory virus [2]. Patients infected with SARS-CoV often present with fevers, and lower respiratory tract illness [3]. This state of *SARS-CoV-2* infection and the resulting disease is known as coronavirus disease 2019, commonly referred to as COVID-19. The onset of symptoms can be sudden and severe and may be accompanied by a dry cough and other flu-like symptoms such as body aches and headaches. The cough sometimes progresses to pneumonia, which can be fatal despite receiving the best care that modern medicine can provide [4]. 

As of July 2021, *SARS-CoV-2* had infected more than 187 million people, resulting in over four million deaths [5]. Of those who recovered after hospital admission, approximately 60% are believed to have ‘long COVID’ [6]. ‘Long COVID’ refers to a condition whereby COVID-19 symptoms persist beyond 3–4 weeks after the initial infection [7,8]. These symptoms include pulmonary, cardiovascular, haematological, neuropsychiatric, renal, endocrine, gastrointestinal, hepatobiliary, and inflammatory sequelae [9]. In a span of less than two years, strategies for dealing with the outbreak and global spread of the virus have transitioned from containment to attempted management to prevention by means of vaccination. 

Vitamin D is a well-documented modulator of immune responses. Due to the immense toll of COVID-19 on the immune system, there has been significant interest in the potential of vitamin D to ameliorate or prevent detrimental immune responses. A number of new studies have been published, warranting narrative synthesis to summarise the state of knowledge in this field. This review thus aims to consolidate the evidence surrounding vitamin D in relation to COVID-19, with the intention of gauging the current consensus regarding vitamin D supplementation as a means to treat and/or prevent COVID-19 onset or progression. We provide an overview of the state of knowledge in this area, with reference to key studies and systematic reviews that have been published to date. 

## 2. Virology of *SARS-CoV-2*

In terms of virology, *SARS-CoV-2* is an enveloped, positive sense, single-stranded ribonucleic acid (RNA) virus [10]. The virus is able to spread easily and can mutate to form new strains which are often more virulent and more contagious [11]. The α (alpha) variant isolated in the UK was found to be 75% more transmissible [12] and 61% more fatal [13] compared to the original outbreak, while the Δ (delta) strain isolated in India was found to be even more contagious. The spike protein on the envelope of coronaviruses is responsible for the crown-like appearance of the virion [14]. TMPRSS2, a serine protease found on the host cell primes the spike protein, which then binds to the angiotensin-converting enzyme 2 (ACE2) “receptors” [15]. ACE2 physiologically inactivates angiotensin II and serves as a negative regulator of the renin-angiotensin-aldosterone (RAAS) system [16]. ACE2 is not a receptor in the classical sense where ligands bind and result in a downstream intracellular response. It is an enzyme found on the surface of many different cell types. However, *SARS-CoV-2* uses it as a receptor to mediate entry into epithelial cells. Not surprisingly, this can be quite deleterious to cardiovascular health. Investigations after the 2004 SARS-CoV outbreak revealed that mutations within the receptor binding domain of the spike protein were the key factor in determining how contagious a particular strain was [17]. As compared to the 2004 outbreak, *SARS-CoV-2* is much more contagious [18]. This is attributed to changes in the chemistry of the binding site, which result in easier cleavage of specific viral envelope proteins, allowing easier entry into host cells [17]. Furthermore, post-translational addition of glycans to flank this cleavage site have been speculated to protect it from host immune responses [19]. Virus particles are only able to enter ACE2 expressing cells [20]. In the respiratory tract, *SARS-CoV-2* particles enter directly via type 2 alveoli cells, which are physiologically responsible for the production of surfactant. Binding to ACE2 triggers endocytosis, followed by membrane fusion, allowing the uncoated (+)ssRNA to enter the cytoplasm [21]. Once in the cell, the positive sense RNA of the virus uses the host cell translation machinery and resources to produce proteins required for the production of more virions [22]. These host cells are thus unable to fulfil their physiological function, resulting in a reduction in surfactant production and a subsequent reduction in oxygen saturation, increased rate of respiration, and ultimately, respiratory failure [23].

## 3. Pathogenesis/Pathophysiology

A study in mouse models of *SARS-CoV* infections showed that ACE2 was not only crucial as a receptor for viral entry, but also resulted in decreased expression of ACE2, which was directly linked to severe lung failure and adult respiratory distress syndrome (ARDS) [24,25]. Mechanistically, ACE2 downregulation or knockouts have been linked to increased elastase activity (indicating increased stiffness of the lung), decreased blood oxygenation, and thus pulmonary oedema [26]. In addition, aspiration of gastric fluids can also lead to lung injury or symptoms of ARDS [27]. 

In a phenomenon called a ‘cytokine storm’, an overly active immune system has been known to disrupt the delicate balance between clearing the infection and an auto-immune condition. These cytokine storms have been referred to in the context of graft-versus-host disease in transplantations [28], as well as in the H5N1 avian influenza virus infection [29]. Acute lung injury, which results from infection or insult to the lungs, causes resident lung cells to release chemoattractants, which recruit circulating neutrophils to infiltrate the epithelium and airspaces [30]. Infiltration increases the permeability of the epithelial and endothelial surfaces by means of toxic proteases, peptides and oxidants. This results in apoptosis of endothelial and epithelial cells [31]. However, even in the absence of apoptosis, the inflammatory effects of neutrophils within the epithelium are a significant component of lung injury [31]. This inflammation reduces fluid exchange and surfactant production, leading to oedema [32]. 

The initial infection induces various protective inflammatory responses in the host, including an increase in the expression of pro-inflammatory cytokines such as interferon (IFN)-γ, interleukin (IL)-1, IL-6 and IL-12, as well as in chemokines such as C-C motif chemokine ligands (CCL) including CCL-2 (also known as monocyte chemoattractant protein-1) and CCL-5 (also known as RANTES), as well as C-X-C motif chemokine ligands (CXCL) including CXCL2 (also known as macrophage inflammatory protein-2 α) and CXCL8 (also known as IL-8) [33,34]. An increase in cytokine production leads to increased recruitment of immune cells to the area and thus greater compromise to the structural integrity of the vasculature and epithelia [31]. These cytokines and chemokines cause T cells to favour a Th1 phenotype to mount a full pro-inflammatory response. The delayed, excessive production of these cytokines is pathological. Clinically, it was observed that SARS patients in the intensive care unit (ICU) had markedly higher levels of these markers, as well as tumour necrosis factor (TNFα), a pyrogenic pro-inflammatory mediator that is associated with infection and autoimmune disorders. In addition, there were reduced levels of the anti-inflammatory marker, IL-10 [35]. Histology of patients who died from previous coronaviruses such as *SARS-CoV* showed increased neutrophils and macrophages in lung infiltrates, in addition to increased neutrophils and monocytes and reduced CD4^+^ and CD8^+^ cell counts in the periphery [36,37]. As a result of the Th-1 shift and excessive inflammation in COVID-19 patients, ARDS often leads to cardiopulmonary disorders, multiple organ failure, and eventually death [38]. 

## 4. Vitamin D and the Immune System

Vitamin D has been classically linked to calcium absorption and bone health. However, over the last decade, the key role of vitamin D in inflammation and immunoregulation has been increasingly recognised. Despite this critical role, the prevalence of vitamin D deficiency remains widespread, with 25-hydroxyvitamin D (25(OH)D) levels below 50 nmol/L (the common threshold for defining vitamin D deficiency [39]) affecting approximately 25% of US and Australian populations, as well as nearly 40% of Canadian and European populations [40,41]. These deficiency rates are largely attributed to the rising prevalence of obesity and sedentary indoor lifestyles, as well as sunscreen use and sun avoidance from fears of skin cancer.

Vitamin D can be derived from supplements in the form of vitamin D_2_ (ergocalciferol) or D_3_ (cholecalciferol) with limited amounts available via dietary sources including oily fish, egg yolk and fortified dairy [42]. Nevertheless, sun exposure remains the main source of vitamin D, whereby skin exposure to ultraviolet B (UVB) radiation results in the proteolytic cleavage of 7-dihydrocholesterol for conversion into pre-vitamin D_3_ [43]. Pre-vitamin D_3_ is stabilised by spontaneous isomerisation and ejected from the cell in the dermis/epidermis before being transported systemically [43]. This compound, however, is biologically inert and undergoes a series of hydroxylations, first by 25-hydroxylase (*CYP2R1*) in the liver, then by 1α,25-hydroxylase (*CYP27B1*) in the kidney, to form the immunologically active form of vitamin D, 1α,25-dihydroxyvitamin D (1,25(OH)_2_D_3_ or calcitriol) [44,45]. Calcitriol has several functions that regulate the response of both the innate and adaptive immune systems. For instance, calcitriol modulates cytokine and chemokine expression and decreases IL-12 production in dendritic cells as well as IL-2 and IFNγ production by CD4^+^ T cells [46].

Vitamin D exerts its effects by binding to a nuclear vitamin D receptor (VDR), which is expressed in various immune cells, with particularly high levels in dendritic cells (antigen-presenting cells), macrophages, and T (e.g., CD4^+^ and CD8^+^) and B lymphocytes [47,48]. As a steroid hormone, vitamin D is able to cross lipid membranes, and interact directly with its nuclear receptor. This binding action leads to transcriptional regulation of target genes, including those coding for inflammatory mediators and proteins required to alter phenotypes of immune cells [49]. However, the immunomodulatory effects of vitamin D signalling are determined by phenotypes of the target immune cells. For example, naïve T cells express VDRs in basal concentrations, but this is increased dramatically upon activation [50,51]. In monocytes, however, differentiated monocytes (macrophages/dendritic cells) express fewer VDRs [52]. Vitamin D is also involved in several signalling pathways that are activated in a non-genomic manner by calcitriol [53]. 

Vitamin D has also been said to affect protein synthesis, where it interacts with proteins other than the VDR that are involved in the folding of newly synthesised glycoproteins [54]. It has also been postulated that supplementation of vitamin D alters pathways involved in histone and chromatin modifications in a manner distinct from VDR activity [55]. In a novel biosynthetic pathway mediated by *CYP11A1*, vitamin D is also activated by hydroxylation at different sites, resulting in the formation of different non-calcaemic hydroxyderivatives that act with different potency on the VDR [56]. The site of hydroxylation determines the action of the derivative, in that they may act as partial or selective agonists of the VDR, but as full agonists of alternative nuclear receptors [57]. These *CYP11A1*-derived hydroxyderivatives, in addition to classical calcitriol, exert important anti-inflammatory and anti-oxidative effects involving downregulation of nuclear factor *kappa*-B (NF-κΒ) and inverse agonism on retinoic acid-related orphan receptors (RORγ) resulting in diminished Th17 responses. Anti-oxidative and reparative responses have also been demonstrated, involving activation of nuclear factor erythroid 2p45-related factor 2 and p53 [58]. Importantly, vitamin D delivered orally is not recognized by *CYP11A1*. Hence, parenteral delivery of vitamin D would be required to bypass the liver and produce *CYP11A1*-derived hydroxyderivatives, with corresponding anti-oxidative and cytokine storm-suppressing effects, potentially mitigating multi-organ damage and death from COVID-19 [58].

### 4.1. Vitamin D in Innate Immunity

One aspect of innate immunity influenced by calcitriol is the enhancement of phagocytotic activity of macrophages. Macrophages and monocytes use Toll-like receptors (TLRs) to sense pathogen-associated molecular patterns (PAMPs) on infectious agents, and in turn, phagocytose these agents in what is known as the first line of defence. Calcitriol has been shown to enhance the antimicrobial capabilities of these innate immune cells. 

Immune signalling such as those of IFNγ, STAT-1α, lipopolysaccharide (LPS) and TLRs increases expression of 1α hydroxylase in monocytes. 1α hydroxylase catalyses the formation of 1,25(OH)_2_D_3_, which has been shown to stimulate monocyte differentiation, with cells looking phenotypically like macrophages following 1,25(OH)_2_D_3_ exposure [59]. In macrophages, stimulation with 1,25(OH)_2_D_3_ has an anti-inflammatory effect by increasing IL-10 production and decreasing pro-inflammatory markers such as TNFα, IL-1β, IL-6, and cyclooxygenase-2 (COX-2), by means of the mitogen-activated protein kinase (MAPK) phosphatase pathway [60,61].

In addition, while TLR activation results in increased expression of the VDR, 1,25(OH)_2_D_3_ is able to downregulate the expression of miRNA155, which when expressed, reduces anti-inflammatory gene expression [62,63]. Studies have also shown that the ligand-bound VDR is able to modulate the epigenome of immune cells, monocytes in particular [64]. 

In dendritic cells, 1,25(OH)_2_D_3_ binding triggers a shift to a more tolerogenic phenotype, both in cell morphology and expression of surface proteins [65,66]. There is a marked decrease in major histocompatibility complex (MHC) class II receptors as well as CD80 and CD86 co-stimulatory molecules needed to mount an immune response [66]. Simultaneously, there is an increase in CCR-5 and CD40 receptors, favouring an anti-inflammatory environment [67]. Additionally, 1,25(OH)_2_D_3_ increases expression of programmed death-ligand 1 (PD-L1) and TNF, which induces the activity of regulatory T cells—a net anti-inflammatory effect [68,69].

VDRs are also expressed in natural killer (NK) cells and neutrophils [70]. VDR signalling has been shown to enhance neutrophil killing of naïve cells while decreasing the amount of pro-inflammatory cytokines produced by infected neutrophils [71]. Essentially, 1,25(OH)_2_D_3_ optimises neutrophil response while preventing excessive response. 1,25(OH)_2_D_3_ has also been shown to modulate NK cell response by decreasing expression of IFNγ and reducing cytotoxic activity [72,73].

### 4.2. Vitamin D in Adaptive Immunity

The anti-inflammatory effect of 1,25(OH)_2_D_3_ signalling on dendritic cells and macrophages (i.e., downregulation of MHC II and co-stimulatory molecules, reduced pro-inflammatory signalling) modulates the response of T lymphocytes. This decreases proliferation of autoreactive T cells, which have the potential for autoimmune activity, and in some cases, increases proliferation of regulatory T cells (Tregs) [74,75]. Anti-inflammatory cytokines also induce a Th2 phenotype as opposed to Th1 or Th17 [76].

Directly, however, 1,25(OH)_2_D_3_ signalling in T cells is varied depending on the phenotype and state of differentiation. Upon activation, T lymphocytes express higher levels of VDR [77]. Stimulation with 1,25(OH)_2_D_3_ reduces signalling of IL-2 and IFNγ (Th1) and IL-17 and IL-21 (Th17) cytokines [76,78], while acting synergistically with IL-2 to induce and perpetuate a Th2 phenotype [78].

VDR expression in B cells has been shown to induce apoptosis in activated B cells. In addition, 1,25(OH)_2_D_3_ signalling reduces generation of plasma cells and B cells that have undergone antibody class switching, without affecting B cell differentiation itself [79,80]. It has since been speculated that calcitriol has potentially beneficial effects in the context of the role of B cells in autoimmune diseases [70]. Calcitriol also increases anti-inflammatory IL-10 production in B cells and reduces T cell activation by B cells by altering the composition of cell surface markers [81].

More recently, there has been evidence showing that vitamin D and its hydroxyderivatives are able to bind and activate the liver X receptor (LXR) [82]. The LXR has been independently linked to regulating the switch in T cell phenotypes and thus the function of the adaptive immune system [83].

## 5. Vitamin D and COVID-19: Summary of the Evidence

### 5.1. Overview

**Figure 1:**
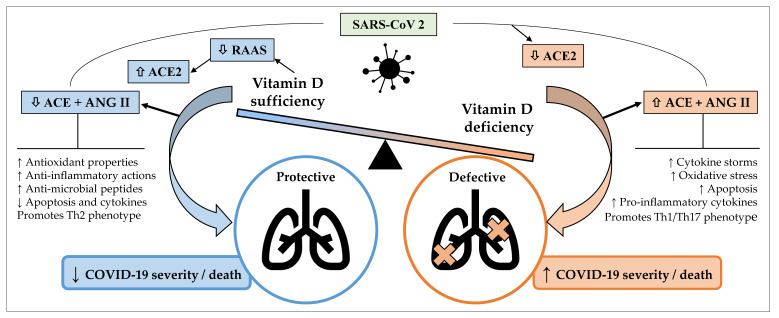
Potential mechanisms by which sufficient serum vitamin D levels may confer protective effects against coronavirus disease 2019 (COVID-19) and acute lung injury, whereas vitamin D deficiency may contribute to a defective immune response against COVID-19 and increased severity and/or mortality. ACE, angiotensin-converting enzyme; ANG, angiotensin; COVID-19, coronavirus disease 2019; RAAS, renin angiotensin aldosterone system; *SARS-CoV-2*, severe acute respiratory syndrome coronavirus 2. Arrows pointing up indicate an increase; arrows pointing down indicate a decrease.

The putative role of vitamin D in the treatment or management of COVID-19 is complex and dynamic. As described above, vitamin D is capable of modulating different aspects of immunity, with potential to influence COVID-19 severity and outcomes. *SARS-CoV-2* infections lead to downregulation of ACE2, causing toxic accumulation of Ang II, which in turn contributes to ARDS. Vitamin D has been found to attenuate the effects of these interactions between *SARS-CoV-2* and the RAAS [84]. As a negative endocrine regulator on the RAAS, vitamin D is able to induce the vasorelaxant ACE2/Ang-(1-7)/Mas receptor axis, which protects against acute lung injury and ARDS (Figure 1) [85]. Vitamin D is a negative regulator of renin biosynthesis and works by binding to the VDR and preventing expression of renin-producing enzymes and proteins [86]. Vitamin D has also been shown to increase the expression of ACE2 [86]. There has been evidence suggesting that a potential cause for the differential presentation of COVID-19 between males and females (higher odds of ICU admissions and death in males) is due to different expressions of ACE2, which results in a heightened and more effective immune response in females [87,88,89]. The influence of ACE2 expression on COVID-19 severity is determined at this stage, where it has been established that ACE2 is expressed in greater concentrations in males than females [90,91]. A study using an animal model found that females post oophorectomy showed increased ACE2 activity compared to before, showing that many of these differences can be attributed to the presence of oestrogen [92]. Essentially, males having increased ACE2 expression and not having the cardioprotective effect of oestrogen results in increased severity of COVID-19. While complex mechanisms for the cardiovascular sequelae of COVID-19 have been proposed [93], that the damage occurs is undeniable [94]. COVID-19 causes the burden on the cardiovascular system to be dramatically increased, a process in which ACE2 and the RAAS have been implicated [93], and those with pre-existing cardiovascular morbidities are at greater risk of complications and mortality [94]. A case analysis of 43 patients in China found that males were more likely to have more severe COVID-19 as opposed to females [95]. Similar differences were observed in the US and Europe [96,97,98].

Other observational studies suggested that different levels of 1,25(OH)_2_D_3_ and VDR expression may be implicated in the differences between males and females [99,100]. Beyond the mechanistic explanation of 1,25(OH)_2_D_3_ signalling altering the immune response, females have the added benefit of vitamin D working synergistically with oestrogen, exerting a protective effect in CD4+ T cells to prevent an autoimmune response [101]. However, describing gender differences in COVID-19 as a result of ACE2 or vitamin D may be simplistic. In a manner that could be highly variable due to individual differences, this could be attributed to fewer morbidities, as oestrogen confers a protective effect in cardiovascular and immunological health [102]. 

Local activation from 25(OH)D_3_ to the active 1,25(OH)_2_D_3_ is of considerable importance in the context of an antiviral response in the respiratory system. VDRs are constitutively expressed, and 1α hydroxylase is present in high levels in macrophages and epithelia, among other cell types [103,104,105,106]. Stimulation of 1,25(OH)_2_D_3_ in lung epithelia has been shown to induce expression of antimicrobial peptides such as cathelicidin 1 and defensin β4, in addition to the production of cathelicidin 1 in large amounts by macrophages, and lower amounts in dendritic cells [63,107]. 1,25(OH)_2_D_3_ and VDR signalling play a similarly significant role in the lung epithelia, and their roles in modulating responses to influenza and respiratory syncytial virus have been characterised [108,109]. It is possible that insufficient vitamin D levels contribute to cytokine storms, inadequate protection from epithelial cell apoptosis, and deficient epithelial cell repair, ultimately making the lungs vulnerable to fatal immune system dysregulation. The evidence that vitamin D is a better and more holistic modulator of the immune system than antibody therapy to prevent the action of cytokines like IL-6 is not surprising [110]. 

It was found that vitamin D and its biologically active hydroxyderivatives are likely to inhibit the action of the TMPRSS2 [111]. This results in blocking of the fusion between the viral spike protein and ACE2, which is required for viral entry into the host cell. The biologically active molecules that prevent this interaction with the serine protease have varying synthetic pathways and carry out different biological functions. Based on computational investigations, 1α,25(OH)_2_D_3_, 25(OH)D_3_, 1α,20S(OH)_2_D_3_, 20S,23R(OH)_2_D_3_, 20S,23S(OH)_2_D_3_ and 1α,20S,23S(OH)_3_D_3_ are promising inhibitors of TMPRSS2 [111]. There has been evidence of vitamin D attenuating damage from pro-inflammatory mediators such as NF-κB and IL-17 in the skin after exposure to UVB [57]. If replicated in the systemic circulation, this mechanism is highly likely to ameliorate the effects of cytokine storms [112]. As mentioned earlier, however, the route of delivery of vitamin D is a key factor that should be considered since this has a profound impact on circulating derivatives of vitamin D [58].

In addition, a study investigating the targets of *SARS-CoV-2* using genomics guided tracing has also confirmed the role of vitamin D in COVID-19. Glinsky [113] explored vitamin D as a putative repressor of ACE2 expression and found that vitamin D appeared to inhibit ACE2 expression in human bronchial smooth muscle cells by means of the VDR and other transcription factors. Of the 332 genes coding for the prey proteins of *SARS-CoV-2*, vitamin D affects the expression of 84 (25%). These prey proteins carry out a host of cellular functions which are disrupted by infection. This suggests that, in addition to inhibiting the expression of ACE-2, vitamin D is able to disrupt the function of 19 out of 27 (70%) *SARS-CoV-2* proteins [113]. While highly convincing, mechanistic studies confirming the role of vitamin D in *SARS-CoV-2* infections using transcriptomic or metabolomic analyses are eagerly awaited. Nevertheless, several observational studies and randomised controlled or open-label trials have shown significant associations between vitamin D and COVID-19 (Table 1). 

### 5.2. Observational Studies

In the context of *SARS-CoV-2* infections, there have been several observational studies investigating the role of vitamin D. A large cross-sectional study examining the geographical distribution of COVID-19 in the US drew links between sunlight exposure as a surrogate measure for vitamin D and outcomes such as disease severity and death from COVID-19 [159]. Similarly, correlation analysis of data from 88 countries found that countries proximal to the equator had lower COVID-19 fatalities compared with distal countries, suggesting a potential link between vitamin D (by proxy of latitude and sunlight exposure) and COVID-19 fatality [114]. In a cohort study of an aging population, it was observed that COVID-19 patients admitted to a hospital in the UK had lower serum levels of vitamin D compared with healthy controls, and there was a noticeable correlation between vitamin D deficiency and needing increased care or ventilation [115]. Baktash et al. [115] also corroborated findings of vitamin D deficiency being associated with increased incidence of cytokine storms. Similarly, in two studies of 107 and 4314 patients in Switzerland and Chicago, those who tested positive for *SARS-CoV-2* were more likely to have lower circulating 25(OH)D concentrations [116,117]. A retrospective study of 185 patients diagnosed with COVID-19 showed that those with low serum 25(OH)D levels were more likely to have poor outcomes, defined as the need for invasive mechanical ventilation or death [118]. Several studies have shown that receiving cholecalciferol or calcifediol treatment improves outcomes such as ICU admission and mortality [139,149,151]. Others have found associations between vitamin D sufficiency and reduced incidence of COVID-19, but not in outcomes after infection [155,158]. However, some studies have reported contrary, negative results [143,144,147]. Overall, the evidence supports the importance of the relationship between vitamin D and COVID-19, a relationship that requires continued investigation by means of large-scale, nationally representative studies.

### 5.3. Clinical Trials

A randomised prospective open-label study in India of 87 patients with COVID-19 and hypovitaminosis D reported that supplementing vitamin D in addition to standard care improved inflammatory markers significantly [119]. In the patients that received 60,000 IU of daily supplemental vitamin D for eight days, levels of C-reactive protein, lactase dehydrogenase, IL-6, ferritin, as well as neutrophil to lymphocyte ratios showed significant improvement compared to patients receiving no supplements. This was corroborated by a retrospective cohort study where COVID-19 patients with vitamin D deficiency had significantly lower haemoglobin and lymphocyte counts and higher levels of inflammatory markers, including C-reactive protein [120]. In addition, vitamin D deficient patients tended to require oxygen therapy and patients who had not corrected their vitamin D levels six months prior to *SARS-CoV-2* infection were more likely to be diagnosed with pneumonia [120]. A randomised controlled trial (RCT) in Spain [121] investigated the effects of hydroxychloroquine and azithromycin in combination with oral calcifediol in the treatment of COVID-19. While hydroxychloroquine and azithromycin were administered according to standard of care treatment, they reported that sufficient vitamin D levels brought about by calcifediol supplementation was the most apparent factor in determining disease prognosis [121]. Rastogi et al. [123] performed a RCT in India and found that three times as many asymptomatic or mildly symptomatic patients receiving high dose vitamin D (60,000 IU of daily cholecalciferol for 7 days) reaching a therapeutic target of 25(OH)D > 50 ng/mL could attain a negative *SARS-CoV-2* RNA by day 21, compared with vitamin D-deficient placebo recipients. Conversely, another RCT in Brazil reported that among 240 hospitalized patients with COVID-19, a single high dose of 200,000 IU of cholecalciferol did not reduce hospital length of stay or affect in-hospital mortality, admission to ICU or need for mechanical ventilation compared with placebo [122]. It should be noted that participants in this study received diverse concomitant medications and were given vitamin D after a relatively long time from symptom onset (mean 10.3 days). Hence, it is unclear whether the null findings may be due to this delay and whether preventive or early vitamin D supplementation may be useful in treating mild or moderate COVID-19. Indeed, in a quasi-randomised trial investigating the possible prevention of infection in 66 aged-care residents in France, it was reported that vitamin D supplementation during or just before COVID-19 was associated with less severe disease and lower mortality rate [124]. Simultaneously, it was also shown that regular vitamin D supplementation prior to infection led to better survival among elderly patients [128]. A randomized controlled trial also showed that daily administration for 2 weeks of 5000 IU but not 1000 IU was reduced recovery time in patients with mild to moderate symptoms [154]. This was also seen from their decreases in BMI and IL-6 levels over time. These studies support the potential for vitamin D supplementation to be used as an adjunct therapy to improve clinical outcomes in COVID-19 [125].

## 6. Limitations and Future Directions

The progress of the field is, to a great extent, limited by the rapidly evolving nature of the virus as well as its emerging effects on the immune system which are not yet fully understood. The phenomenon of more contagious or virulent strains of the virus causing adverse outcomes even in younger populations that are thought to be more immunologically resilient, calls for re-examination of what little is known. Current evidence, while overwhelmingly supporting the hypothesis implicating vitamin D, is not absolute, with some groups reporting no relationship between vitamin D and COVID-19 once other confounding factors are considered [160]. In addition, the root cause of the lack of vitamin D and by extension, the immune dysfunction seen in COVID-19, is still being debated. The notion that diabetes and obesity (both concomitant with vitamin D deficient states) result in the increased fatality from COVID-19, rather than lack of vitamin D itself, has also been proposed and provides a plausible explanation [161]. Others argue the “healthy user effect”, where healthy people who eat and exercise well spend more time outdoors and thus have higher vitamin D levels. This supports the correlations between vitamin D deficiency and poor outcomes after COVID-19, but not causation. Across the clinical trials reviewed here, it is apparent that there is substantial clinical and methodological heterogeneity, mainly owing to the different supplementation regimens and outcomes, as well as varied vitamin D status of participants. Together, these discrepancies preclude definitive conclusions and highlight the need for well-designed, adequately powered trials to determine the role of vitamin D in COVID-19. As noted in a recent Cochrane review [126], there are currently 21 ongoing trials that may shed some light on this topic in the near future. 

In addition, it is important to acknowledge data limitations in observational studies. There are numerous reasons for the conclusions drawn from analysis of the dataset not being representative, and thus not being applicable. These include insufficient power, having a sample that is not representative of the population, the potential for false positives or negatives, or even unique circumstances that were overlooked and not accounted for in the multivariate analysis. 

While there has been no preference in the literature regarding whether vitamin D is obtained via UVB exposure or supplementally, it is important to consider seasonal changes in physiological vitamin D synthesis. As a fat-soluble steroid hormone, vitamin D can be stored in adipose reservoirs for weeks at a time [162]. Supplementation should therefore take the pharmacokinetics of vitamin D into account. It should also be noted that studies demonstrating positive effects of vitamin D supplementation recommend treatment to restore levels to normal [159], whereas supraphysiological concentrations are not recommended. There have also been recommendations to pre-emptively treat low vitamin D levels as it reduces the severity of disease after infection. This needs to be considered and discussed further in the context of other risk factors such as aging and age-related deterioration of health [163]. Overall, there remains a great deal of work needed to evaluate the immunological effects of the mutant strains of *SARS-CoV-2*. Moreover, the mechanistic pathways underlying the cellular and molecular functions of vitamin D concentrations in the context of COVID-19 await further study. 

## 7. Conclusions

In summary, current evidence supports the links between vitamin D and COVID-19 and the benefits of vitamin D supplementation for managing or treating this condition. Most of the literature reports improved COVID-19 prognosis and outcomes with sufficient vitamin D concentrations, with or without supplementation, with some reporting no significant differences based on vitamin D levels and/or no improvements following supplementation. Some even report a decreased incidence of infection as a result of prior supplementation. Future research should focus on establishing the mechanism/s for this link, as well as optimising treatment doses for maximum benefit to patients once infected. In the meantime, vitamin D deficiency should be corrected wherever possible since vitamin D supplementation is safe and the potential for toxicity is strongly outweighed by the potential benefits in relation to protection from COVID-19.

## Figures and Tables

**Table 1:** Characteristics of studies examining the links between vitamin D and COVID-19 *.

Author, Year, Country [Ref]	Study Design	Sample Size	Main Findings
Whittemore et al., 2020 [114]	Correlation analysis of data	88 countries	Countries closer to the equator had lower COVID-19 fatalities than countries further away; 16% of this effect was attributed to latitude.
Baktash et al., 2021, UK [115]	Cohort study	105	Vitamin D deficiency was associated with needing more care, and with the occurrence of cytokine storms.
D’Avolio et al., 2020, Switzerland [116]	Retrospective cohort study	107	People who tested positive for *SARS-CoV-2* had lower levels of 25(OH)D.
Meltzer et al., 2020, USA [117]	Retrospective cohort study	4314	Deficient vitamin D status was linked to increased risk of COVID-19.
Radujkovic et al., 2020, Germany [118]	Retrospective study	185	Vitamin D deficiency was associated with a higher risk of invasive mechanical ventilation or death.
Lakkireddy et al., 2021, India [119]	Randomised prospective open-label study	87	Vitamin D supplementation in those with hypovitaminosis D improved inflammatory markers.
Ünsal et al., 2021, Turkey [120]	Retrospective cohort study	56	Vitamin D deficient COVID-19 patients had significantly lower haemoglobin and lymphocyte counts and higher levels of inflammatory markers.
Entrenas Castillo et al., 2020, Spain [121]	Parallel pilot randomized open-label trial *	76	High dose calcifediol reduced the severity of COVID-19 in patients requiring ICU admission.
Murai et al., 2021, Brazil [122]	Multi-centre parallel double-blind RCT	240	Single high dose cholecalciferol did not reduce hospital stay, mortality, admission to ICU or need for ventilation compared with placebo.
Rastogi et al., 2020, India [123]	RCT	40	Compared with vitamin D deficient individuals, a greater proportion of asymptomatic or mildly symptomatic patients on high dose vitamin D reaching 25(OH)D >50 ng/ml achieved a negative *SARS-CoV-2* RNA at ≤ 21 days.
Annweiler et al., 2020, France [124]	Quasi-randomised trial	66	Vitamin D supplementation just before or during COVID-19 in an aged population reduced disease severity and mortality rate.
Munshi et al., 2021, [125]	Systematic review and meta-analysis	6 retrospective articles	Vitamin D levels could be a useful prognostic indicator of COVID-19 outcomes.
Stroehlein et al., 2021 [126]	Living Cochrane systematic review	3 RCTs	Current evidence is insufficient to conclusively determine the benefits or harms of vitamin D supplementation as a treatment for COVID-19.
Annweiler et al., 2020, France [127]	Open-label, multicenter, superiority RCT		Single dose of 50,000 IU of vitamin D vs Single dose of 200,000 IU of vitamin D in COVID-19 positive patients showing at least one sign of increased risk.Results pending.
Annweiler et al., 2020, France [128]	Quasi-experimental study	77	Regular vitamin D supplementation prior to COVID-19 led to improved mortality in elderly patients at three months follow-up.
Pereira et al., 2020 [129]	Systematic review and meta-analysis	21 studies	Correlation between severely ill COVID-19 patients and low vitamin D levels [130], possibly explained by 25(OH)D being inversely associated with pro-inflammatory cytokines, such as IL-6, increase in CRP, and cardiac insufficiency, which relate to severity of COVID-19 and to its unfavorable outcomes [131]. Despite the correlation between higher vitamin D levels, immune defenses, and favorable prognoses in other viral infections [132], no causal relationship has been established between 25(OH)D deficiency and vulnerability to infection by COVID-19, by testing and blood vitamin D test in SARS-CoV-2 patients.
Tan et al., 2020, Singapore [133]	Cohort Study	43	Patients were administered 1000 IU/d oral vitamin D3, 150 mg/d oral magnesium, and 500 mcg/d oral vitamin B12 upon admission if they did not require oxygen therapy. After correcting for demographics and hypertension, patients who received DMB had significantly less deterioration to the point of requiring oxygen therapy or intensive care support.
Meltzer et al., 2020, USA [134]	Retrospective cohort study	499	Other than age and non-white race, vitamin D deficient status was correlated with the incidence of COVID-19. However, the dose of vitamin D supplementation was not significantly correlated with the likelihood of testing positive to COVID-19.
Kaufman et al., 2020, USA [135]	Retrospective observational analysis	191,779	*SARS-CoV-2* incidence was higher in the patients with deficient 25(OH)D scores than in patients with adequate values and those with levels ≥55 ng/mL.
Jain et al., 2020, India [136]	Observational study	154	Mean vitamin D levels were significantly higher in asymptomatic patients than severely ill. Prevalence of vitamin D deficiency was significantly higher in the severely ill. Of 154 patients, 90 were deficient in vitamin D (29 asymptomatic; 61 severely ill). Serum levels of inflammatory markers, inflammatory response and fatality rate were higher in vitamin D deficient patients (21% vs 3.1%). Vitamin D level was markedly low in severe COVID-19 patients.
Carpagnano et al., 2020, Italy [137]	Retrospective observational study	42	After 10 days of hospitalization, patients with severe vitamin D deficiency had a 50% mortality probability, while those with insufficiency or moderate deficiency had a 5% mortality risk. COVID-19 patients with acute respiratory failure treated in RICU were found to have a high prevalence of hypovitaminosis D, which correlated with a high mortality risk.
Padhi et al., 2020, India [138]	Observational study	NA	Mean vitamin D levels reported in different states and territories correlated inversely with mortality data collected via government statistics.
Ling et al., 2020, UK [139]	Retrospective observational study	444	Administration of cholecalciferol booster was correlated with a reduced risk of COVID-19 mortality
De Smet et al., 2021, Belgium [140]	Retrospective observational trial	186	Vitamin D deficiency on admission was associated with mortality, independently of age, chronic lung disease, and the extent of lung damage seen from chest CT severity score.
Karahan et al., 2021, Turkey [141]	Retrospective observational study	149	Serum 25(OH)D deficiency was associated with increased mortality in COVID-19 patients.
AlSafar et al., 2021, UAE, [142]	Retrospective observational study	464	After ruling out sex as a predictor for COVID-19 severity or mortality, 25(OH)D levels below 12 ng/mL were significantly correlated with increased risk of severe illness and mortality.
Orchard et al., 2021, UK [143]	Cohort study	646	No significant correlation reported between low vitamin D levels and severity of COVID-19 or mortality.
Osman et al., 2021, Oman [144]	Observational cohort study	445	While no correlation between vitamin D and disease severity and progression was observed, there was an association between hypocalcaemia and COVID-19 severity. The relationship between calcium and vitamin D is also acknowledged.
Diaz-Curiel et al., 2021, Spain [145]	Retrospective observational study	1549	Vitamin D deficiency was correlated with an increased risk of hospital admission and critical care, but not mortality.
Angelidi et al., 2021, USA [146]	Retrospective cohort study	144	Mortality in hospital and need for mechanical ventilation were inversely correlated with serum vitamin D level
Jevalikar et al., 2021, Indian [147]	Prospective observational study	410	No association between vitamin D deficiency and incidence of severe COVID-19, increased oxygen requirement, ICU admissions or mortality. In vitamin D deficient patients who received cholecalciferol treatment, there was no significant improvement in outcome.
Alcala-Diaz et al., 2021, Spain [148]	Retrospective, multicentre, non-randomised cohort study	537	Calcifediol treatment after COVID-19 diagnosis was significantly associated with reduced 30-day mortality
Cangiano et al., 2020, Italy [149]	Observational study	157	Mortality in COVID-19 patients was found to be inversely associated with vitamin D supplementation
Fasano et al., 2020, Italy [150]	Cohort study	1486	Among Parkinson’s Disease patients, COVID-19 incidence was greater among those who were younger, obese and those with COPD. It was less likely among patients who had vitamin D supplementation.
Giannini et al., 2021, Italy [151]	Retrospective study	91	Two doses of 200,000 IU of vitamin D administered on consecutive days can improve outcomes (ICU admission, mortality) in patients with comorbidities.
Israel et al., 2021, Israel [152]	Case-control study	60,039	The only statins that exerted a protective effect in COVID-19 were those that increased levels of 25(OH)D, such as rosuvastatin.
Loucera et al., 2021, Andalusia [153]	Retrospective survival study	16,401	There was significant reduction in mortality after administration of vitamin D (calcifediol) 15–30 days before hospitalisation
Sabico et al., 2021, Saudi Arabia [154]	RCT	69	5000 IU, but not 1000 IU, daily administration of vitamin D for 2 weeks reduced recovery time for COVID-19 patients with mild to moderate symptoms. Both groups showed decreases in levels of IL-6 and BMI over time.
Oristell et al., 2021, Catalonia [155]	Population based cohort study	108,343	Reduced serum 25(OH)D levels were associated with an increased incidence of infection rather than disease severity or mortality. Groups compared were supplemented vitamin D sufficient versus non-supplemented vitamin D deficient patients who contracted COVID-19
Ohaegbulam et al., 2020, USA [156]	Clinical case series	4	Patients who received high dose supplementation in the form of cholecalciferol or ergocalciferol had faster recovery, seen from reduced inflammatory markers, comparative lower oxygen requirements and reduced duration of hospital stay.
Nogues et al., 2021, Spain [157]	Observational study	838	In hospitalised COVID-19 patients, treatment with calcifediol significantly reduced mortality and admission to ICU
Ma et al. 2021, UK [158]	Prospective study	8297	Habitual vitamin D supplementation was correlated with a reduction in COVID-19 incidence.

**Abbreviations:** 25(OH)D, 25-hydroxyvitamin D; BMI, body mass index; COPD, chronic obstructive pulmonary disorder; COVID-19, coronavirus disease 2019; CRP, C-reactive protein; CT, computed tomography; DMB, combination of vitamin D3, magnesium and vitamin B12; ICU, intensive care unit; IL, interleukin; IU, international units; RCT, randomised controlled trial; RICU, respiratory intensive care unit; SARS-CoV-2, coronavirus severe acute respiratory syndrome. This list is representative of most observational and clinical studies conducted, not exhaustive. * open-label for hydroxychloroquine and azithromycin, double-blind for vitamin D.

## Data Availability

Not applicable.
